# Galaxy Evolution with Manifold Learning

**DOI:** 10.3390/e28030288

**Published:** 2026-03-03

**Authors:** Tsutomu T. Takeuchi, Suchetha Cooray, Ryusei R. Kano

**Affiliations:** 1 Division of Particle and Astrophysical Science, Nagoya University, Furo-cho, Chikusa-ku, Nagoya 464-8602, Aichi, Japan; kano.ryusei.z5@s.mail.nagoya-u.ac.jp; 2 The Research Center for Statistical Machine Learning, The Institute of Statistical Mathematics, 10-3 Midori-cho, Tachikawa 190-8562, Tokyo, Japan; 3 Kavli Institute Particle Astrophysics and Cosmology, Stanford University, Physics and Astrophysics Building (PAB), 452 Lomita Mall, Stanford, CA 94305, USA; cooraysuchetha@gmail.com; 4 The Institute for Astronomy (IfA), School of Physics and Astronomy, The University of Edinburgh, Royal Observatory, Edinburgh EH9 3HJ, UK

**Keywords:** galaxy evolution, galaxy formation, stellar evolution, star formation rate, stellar mass, multiwavelength luminosity, manifold learning

## Abstract

Matter in the early Universe was nearly uniform, and galaxies emerged through the gravitational growth of small primordial density fluctuations. Astrophysics has been trying to unveil the complex physical phenomena that have caused the formation and evolution of galaxies throughout the 13-billion-year history of the Universe using the first principles of physics. However, since present-day astrophysical big data contain more than 100 explanatory variables, such a conventional methodology faces limits in dealing with such data. We, instead, elucidate the physics of galaxy evolution by applying manifold learning, one of the latest methods of data science, to a feature space spanned by galaxy luminosities and cosmic time. We discovered a low-dimensional nonlinear structure of data points in this space, referred to as the galaxy manifold. We found that the galaxy evolution in the ultraviolet–optical–near-infrared luminosity space is well described by two parameters, star formation and stellar mass evolution, on the manifold. We also discuss a possible way to connect the manifold coordinates to physical quantities.

## 1. Introduction

### 1.1. Galaxy Evolution in the Era of Large Galaxy Surveys

A galaxy is an extremely massive astrophysical system composed of stars, interstellar medium (a multi-phase fluid of gas and dust), and dark matter. Within the observable Universe, there are on the order of several hundred billion galaxies. The Universe was born approximately 13.8 billion years ago, and in its early stages, matter was distributed in an almost uniform manner, with no astrophysical objects such as galaxies. In other words, galaxies are astrophysical objects that formed and have dynamically evolved over cosmic time into their present-day appearance. In addition to the standard paradigm based on primordial density fluctuations, alternative scenarios for structure formation have also been proposed. For example, models involving topological defects arising from the symmetry breaking of fundamental fields in the early Universe have been discussed in the literature (see e.g., [1]). While the present work adopts the standard framework of structure formation, our methodology is not restricted to any specific physical origin of structure and can in principle be applied to galaxy populations produced under different formation scenarios. Time evolution is intrinsic to the nature of galaxies, and studies of galaxy formation and evolution that aim to quantify this time dependence have remained central to galaxy research for more than half a century.

Attempts to explain galaxy evolution quantitatively from physical laws began in the 1970s. Under the assumption that a galaxy forms from a single massive gas cloud, theoretical frameworks were developed to describe the history of star formation and the associated heavy-element nucleosynthesis. Although this line of research was essentially completed as a coherent theoretical framework in the early 1980s by Tinsley [2], it did not mark the end of studies of galaxy evolution. At the same time, advances in cosmology revealed that galaxies grow through mergers. This demonstrated that galaxy evolution is a highly complex process that depends strongly on the surrounding galaxy density and gas density. Thus, it became clear that galaxy evolution is a highly intricate process that depends sensitively on the environment in which a galaxy resides, such as the density of neighboring galaxies and the ambient gas density. A symbolic representation of the equations describing galaxy evolution can be written as follows:

(1) $$\begin{array}{c} \begin{array}{cc} \mathrm{SFR}(t) & =f_{1}\left(\mathrm{SFR},M_{*},M_{\mathrm{mol}},M_{\mathrm{HI}},M_{\mathrm{dust}},M_{\mathrm{halo}},\delta_{\mathrm{gal}},\ldots \right),\\ M_{*}\left(t\right) & =f_{2}\left(\mathrm{SFR},M_{*},M_{\mathrm{mol}},M_{\mathrm{HI}},M_{\mathrm{dust}},M_{\mathrm{halo}},\delta_{\mathrm{gal}},\ldots \right),\\ M_{\mathrm{mol}}\left(t\right) & =f_{3}\left(\mathrm{SFR},M_{*},M_{\mathrm{mol}},M_{\mathrm{HI}},M_{\mathrm{dust}},M_{\mathrm{halo}},\delta_{\mathrm{gal}},\ldots \right),\\ M_{\mathrm{HI}}\left(t\right) & =f_{4}\left(\mathrm{SFR},M_{*},M_{\mathrm{mol}},M_{\mathrm{HI}},M_{\mathrm{dust}},M_{\mathrm{halo}},\delta_{\mathrm{gal}},\ldots \right),\\ M_{\mathrm{dust}}\left(t\right) & =f_{5}\left(\mathrm{SFR},M_{*},M_{\mathrm{mol}},M_{\mathrm{HI}},M_{\mathrm{dust}},M_{\mathrm{halo}},\delta_{\mathrm{gal}},\ldots \right),\\ M_{\mathrm{halo}}\left(t\right) & =f_{6}\left(\mathrm{SFR},M_{*},M_{\mathrm{mol}},M_{\mathrm{HI}},M_{\mathrm{dust}},M_{\mathrm{halo}},\delta_{\mathrm{gal}},\ldots \right),\\ \delta_{\mathrm{gal}}\left(t\right) & =f_{7}\left(\mathrm{SFR},M_{*},M_{\mathrm{mol}},M_{\mathrm{HI}},M_{\mathrm{dust}},M_{\mathrm{halo}},\delta_{\mathrm{gal}},\ldots \right),\\ \vdots \end{array} \end{array}$$

Here, SFR(t), $M_{*}\left(t\right)$, $M_{\mathrm{mol}}\left(t\right)$, $M_{\mathrm{HI}}\left(t\right)$, $M_{\mathrm{dust}}\left(t\right)$, $M_{\mathrm{halo}}\left(t\right)$, and δ(t) denote, respectively, the star formation rate, stellar mass, molecular gas mass, atomic hydrogen gas mass, dust mass, dark matter halo mass, and the local galaxy overdensity at time *t*. The variables appearing on the right-hand side are written symbolically, indicating that each quantity depends on the entire past history of all variables.

In order to formulate galaxy evolution, it is necessary to determine such a large system of equations. Astrophysicists have traditionally constructed governing equations based on first-principles physics; however, once the dimensionality of the parameter space exceeds about ten, such approaches become impractical. From the 1970s through the mid-1980s, classical multivariate analysis techniques, such as principal component analysis (PCA), were employed to connect galaxy physical properties in high-dimensional spaces. As a result, various (log-)linear relations, known as galaxy scaling relations, were discovered. Efforts to unify these scaling relations and identify fundamental relationships led to the concept of the “galaxy manifold”  [3,4] (We stress that, throughout this paper, the term “galaxy manifold” refers to a low-dimensional nonlinear structure embedded in the observational data space (e.g., multi-wavelength luminosity space). It is important to emphasize that this usage is purely in the sense of data geometry and should not be confused with spacetime manifolds in general relativity. In particular, the manifold considered here is a Riemannian manifold constructed from observational feature space. It does not represent a physical spacetime, nor does it involve a pseudo-Riemannian metric of Lorentzian signature.). However, classical PCA is limited to linear relations; while it remains useful for exploratory studies of (log-)linear galaxy relations, the galaxy manifold concept remained highly restricted and was largely forgotten for some time [5,6,7]. With the advent of 21st-century galaxy surveys, hundreds of physical quantities are now available for hundreds of millions of galaxies, constituting a quintessential example of big data in both quality and quantity. The feature space describing galaxies now exceeds 100 dimensions. Consequently, characterizing galaxy evolution is no longer possible using traditional approaches based on physical intuition alone, and fundamentally new methodologies based on entirely different conceptual frameworks are required.

Motivated by these limitations, we initiated a study of galaxy evolution based on alternative, modern methodologies [8]. Specifically, Siudek et al. [8] constructed a 13-dimensional feature space consisting of luminosities at 12 wavelengths spanning from the ultraviolet to the near-infrared range (wavelength range λ=150nm−2.2μm), together with the luminosity at each cosmic age, and we applied the Fisher EM algorithm (FEM: [9]), an unsupervised machine learning method. As a result, FEM successfully discovered, without any arbitrary sample selection, the relation between total stellar mass $M_{*}$ and the star formation rate (SFR), known as the star-forming galaxy main sequence (see Figure 7 of [8]). Furthermore, FEM revealed that the main star-forming sequence is continuously connected, above a certain total stellar mass, to a sequence of galaxies in which star formation has ceased. This structure is inconsistent with the hypothesis that galaxy star formation is quenched abruptly, leading to a discontinuous transition to quiescent galaxies, and can only be uncovered by fully exploiting the information contained in the multi-wavelength luminosity space. This continuous distribution of galaxies represents one projection of the galaxy manifold that encapsulates the fundamental aspects of galaxy evolution. Because of its intrinsically nonlinear structure, the galaxy manifold in multi-wavelength luminosity space could not have been discovered in earlier studies based on classical PCA.

However, astrophysical research cannot be satisfied with a merely quantitative description of the galaxy manifold. It is essential to achieve a complete understanding of its structure and to elucidate its dependence on the (presumably small number of) parameters that govern the physics of galaxy evolution. Achieving this further objective requires more sophisticated methodologies.

### 1.2. Galaxy Manifold in Multi-Wavelength Luminosity Space

The star formation rate (SFR) is defined as the amount of stellar mass formed per unit time and is measured in units of $\left[\mathrm{solar}\mathrm{mass}\;{\mathrm{yr}}^{-1}\right]$ (denoted as $\left[M_{⊙}\;{\mathrm{yr}}^{-1}\right]$). The time evolution of the SFR is referred to as the star formation history, which constitutes one of the most important factors governing galaxy evolution. At wavelengths from the ultraviolet to the near-infrared, the emission spectrum of a galaxy is dominated by contributions from stars and gas. The temperature and lifetime of a star depend strongly on its mass: More massive stars are brighter and hotter, but they have shorter lifetimes. Hot stars emit copiously in the ultraviolet, whereas cool stars are faint in the ultraviolet and radiate primarily in the near-infrared range. Quantitatively, let $\tau_{\mathrm{MS}}$ denote the time during which a star remains on the main sequence, the phase of stable and steady nuclear fusion; *T* the stellar surface temperature; and *L* the stellar luminosity. These quantities can be approximated as

(2) $$\begin{array}{cc} \tau_{\mathrm{MS}} & \propto M^{-2.5}\;, \end{array}$$

(3) $$\begin{array}{cc} L & \propto M^{3.5}\; \end{array}$$

(4) $$\begin{array}{cc} L & \propto T^{4} \end{array}$$

(e.g., [10,11]). As a consequence, higher-temperature stars exhaust their lifetimes earlier, and the star formation history is therefore directly imprinted on the galaxy spectrum. In other words, the star formation history is expected to manifest itself appropriately in the space spanned by the multi-wavelength (band) luminosities of galaxies.

In traditional astronomy, galaxy evolution in multi-wavelength luminosity space has often been characterized using ratios of luminosities at different wavelengths. In astronomy, such ratios are referred to as colors. Consider a pair of monochromatic luminosities, $L_{\lambda_{1}}$ and $L_{\lambda_{2}}$ at two different wavelengths $\lambda_{1}$ and $\lambda_{2}$$\left(\lambda_{1}<\lambda_{2}\right)$. If $L_{\lambda_{1}}<L_{\lambda_{2}}$, the object is described as “red”, whereas if $L_{\lambda_{1}}>L_{\lambda_{2}}$, it is described as “blue.” When the relationship between galaxy luminosity (absolute magnitude) (In optical astronomy (including ultraviolet and near-infrared), when the luminosity of an object is denoted by *L*,

(5) $$\begin{array}{c} M\equiv -2.5\log_{10}L+\mathrm{constant}\;\mathrm{independent}\;\mathrm{of}\;\mathrm{the}\;\mathrm{distance}\;\mathrm{to}\;\mathrm{the}\;\mathrm{galaxy} \end{array}$$

is defined as the absolute magnitude, which is widely used as a proxy for luminosity. The precise definition of magnitude is given in Appendix B) and color is plotted (a color–magnitude diagram), two distinct sequences clearly emerge. This phenomenon is known as the bimodality of galaxy colors. Specifically, a tight sequence of red galaxies (the red sequence) and a more extended sequence of blue galaxies (the blue cloud) are universally observed. Because relatively few galaxies occupy the region between the red sequence and the blue cloud, this region is sometimes referred to as the green valley (e.g., [12]). Galaxies in the blue cloud exhibit active star formation and contain short-lived, high-temperature massive stars, whereas galaxies in the red sequence have ceased star formation and are dominated by low-mass, low-temperature stars. Galaxy evolution is generally thought to proceed from the blue cloud to the red sequence; however, the mechanism by which this transition occurs has long remained an unresolved problem (e.g., [13]). Recent studies have suggested that the region between the blue cloud and the red sequence is not discontinuous but instead forms a continuously connected structure in a three-dimensional space defined by color–color–absolute magnitude (e.g., [14]).

However, conventional approaches to evaluating galaxy evolution based on colors suffer from several inherent problems. A common feature of all astronomical survey data is a bias whereby the data include only objects brighter than the detection limit of the observing instrument. If the magnitude of an object at frequency ν is denoted by $m_{\nu }$, then only objects satisfying $m_{\nu }<m_{\nu }^{\lim}$ are included in the observational data. This bias is known as the magnitude selection effect. As noted above, because color is defined as a ratio of luminosities at two wavelengths, selection effects manifest themselves in a highly intricate manner, making it nearly impossible to verify simple completeness. In astronomical survey data, a dataset is said to be complete when all objects down to the detection limit of the instrument are detected without omission. Studies of galaxy evolution based on color–magnitude diagrams have therefore been plagued by confusion, as these complex selection effects cannot be disentangled from intrinsic physical properties. However, because colors are ratios of luminosities, it is also possible to return to a discussion in the original multi-dimensional space spanned by multi-wavelength luminosities (absolute magnitudes). This approach has the advantage that selection effects can be evaluated in a more direct manner. For example, the bimodality observed in color–magnitude diagrams should correspond to peak structures in the original multi-dimensional luminosity space. We therefore focus on the structures formed by galaxies in the high-dimensional space of multi-wavelength luminosities.

The galaxy manifold discovered by Siudek et al. [8] exhibits a nonlinear structure. Even more remarkably, the spectra of the sample galaxies constituting this galaxy manifold can be distinguished using information from only a small number of broad-band luminosities (luminosities measured over a broad wavelength range are referred to as broad-band luminosities), without requiring combinations of more complex physical quantities. This fact suggests that the multi-wavelength luminosities of galaxies from the ultraviolet and optical to the near-infrared range can be explained by, at most, only a few physical parameters. This represents a new characterization of galaxy evolution that could never have been uncovered using conventional approaches. Motivated by this discovery, we have begun to further explore the galaxy manifold, with the aim of elucidating its dependence on the parameters (presumably no more than a few) that govern the physics of galaxy evolution and ultimately deriving the governing equations of galaxy evolution. To this end, we have turned our attention to manifold learning, a class of methods in modern data science fundamentally different from traditional astronomical methodologies (e.g., [15,16]), and are pursuing further analyses. In this work, we demonstrate that the evolution of galaxies in multi-wavelength luminosity space is effectively confined to a two-dimensional nonlinear manifold, for which its intrinsic coordinates correspond closely to stellar mass and SFR.

Throughout this paper, all calculations involving observational data adopt the cosmological parameters $h=H_{0}/\left(100\;\left[\mathrm{km}\;s^{-1}\;{\mathrm{Mpc}}^{-1}\right]\right)=0.7$, $\Omega_{\Lambda 0}=0.7$, $\Omega_{M0}=0.3$ and curvature parameter $\Omega_{K0}=0$. The meaning of these parameters is explained in Appendix A. We also explain some astrophysical and methodological basics used in this work in Appendix A, Appendix B, Appendix C, Appendix D, Appendix E. These appendices are provided for completeness and are not required for understanding the main results.

## 2. Data

The data used in this study are taken from the Reference Catalog of Galaxy Spectral Energy Distributions (RCSED: [17]). RCSED was constructed by combining the all-sky survey catalog of the ultraviolet space telescope *GALEX*; the catalog of the Sloan Digital Sky Survey (SDSS), a large-scale optical spectroscopic and photometric survey; and the catalog of the UKIRT Infrared Deep Sky Survey (UKIDSS), a wide-area near-infrared survey, using state-of-the-art methods of astronomical spectral analysis. RCSED covers approximately 25% of the sky and contains *k*-corrected (cosmological redshift not only stretches wavelengths but also changes the observed spectral range. Because galaxy spectra exhibit complex wavelength dependence, this introduces a complicated change in observed flux density at a fixed wavelength band. The correction for this effect is referred to as the *k*-correction. See Appendix B for a detailed formulation) photometric data in 11 bands (FUV, NUV, u,g,r,i,z,Y,J,H,K) for several million galaxies, together with information on associated physical quantities. In addition, related information obtained by reprocessing several public datasets has been added to the photometric catalog. The parent object list is based on the spectroscopic sample of non-active galaxies in the redshift range 0.007<z<0.6 from SDSS Data Release 7 (DR7) [18] (specifically, galaxies classified as GAL_EM or GALAXY according to the SDSS spectroscopic classification flag (specclass). That is, galaxies dominated by radiation originating from black holes, such as quasars and type-1 Seyfert galaxies, are excluded from the sample). This dataset contains 800,299 galaxies.

From the full sample, we first selected galaxies with photometric measurements available in all 11 bands, yielding 90,565 galaxies. After removing galaxies with a redshift (in this context, redshift is used as a distance indicator. Details are given in Appendix A) reliability of ≤0.5, the number of galaxies is reduced to 90,460. The substantial reduction relative to the parent sample is mainly due to the limited sky area in which cross-matching with the UKIDSS sample is possible. The primary objective of this study is to discover and quantify universal relations in galaxy luminosity space. In order to avoid magnitude selection effects, we constructed a complete sample based on the SDSS *g*-band magnitude (in astronomy, such a dataset is referred to as volume-limited. Although there is no standard Japanese translation, it denotes a sample that includes, without omission, all objects brighter than a given luminosity $L_{\nu }^{\lim}$ within the volume under consideration).Using the limiting absolute magnitude curve derived from the *g*-band’s limiting magnitude $m_{\mathrm{AB},g}=18.3$ (The subscript AB indicates the AB magnitude system based on a physical definition (see Appendix B)), we determined the absolute magnitude threshold so as to maximize the number of galaxies in the final sample. As a result, we constructed a sample consisting of 27,056 galaxies in the range of $z_{\lim}<0.097$ and $M_{\lim,g}\leq -20.016$. All subsequent analyses are based exclusively on this volume-limited sample.

In manifold learning, preprocessing to optimize the dynamic range of the data values is an important step. In this study, we performed two types of analyses: one in which galaxy luminosities in each band were centered by subtracting the simple mean of the absolute magnitudes and normalized to unit variance and another in which the absolute magnitude values themselves were used without rescaling. The results of these two analyses showed almost no quantitative differences (once each axis was rescaled back to absolute magnitude). Accordingly, in this paper, we present only the results obtained using the absolute magnitudes directly as the data feature space without rescaling. This choice is justified because, for the present sample, stellar emission dominates radiation in all bands, resulting in absolute magnitudes that lie within similar ranges across different bands. However, we note that appropriate normalization will be required in future analyses that incorporate additional physical quantities, such as redshift.

## 3. Methods: Quantification of the Galaxy Manifold via Manifold Learning

### 3.1. Galaxy Manifold in Multi-Wavelength Luminosity Space Revisited

**Figure 1:**
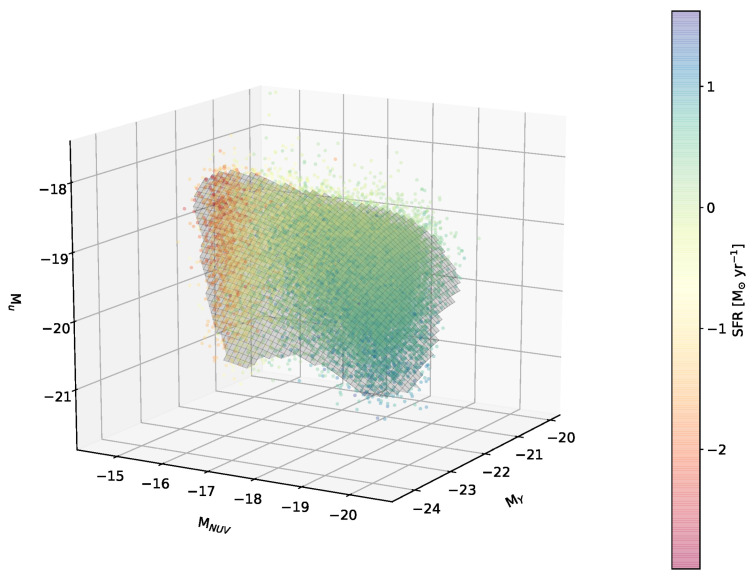
The galaxy manifold discovered in the multi-dimensional feature space of our galaxy sample. Although the original feature space is 11-dimensional, the manifold exhibits only a two-dimensional structure and is essentially embedded in a three-dimensional space defined by ultraviolet, optical, and near-infrared luminosities. Because the manifold has a curved geometry, it cannot be discovered using methods that analyze linear relations, such as classical PCA. The color coding represents the SFR of the sample galaxies.

To perform a basic validation of the sample constructed in Section 2, we first applied FEM to the data, following Siudek et al. [8], and confirmed that a low-dimensional structure formed by galaxies can also be extracted from the 11-dimensional multi-wavelength luminosity space. By analyzing the spatial arrangement of the clusters extracted by FEM, it was shown that this galaxy manifold forms a two-dimensional surface in the feature space of multi-wavelength luminosities. The galaxy manifold derived from RCSED is shown in Figure 1.

Although our galaxy manifold is represented as a low-dimensional subspace embedded in the 11-dimensional luminosity space, it has a curved geometry, making its structure difficult to grasp visually, and further quantitative characterization is nontrivial. The multimodality and scatter observed in classical scaling relations are often the result of non-optimal projections that do not reflect the intrinsic nonlinear structure of the galaxy manifold. As noted in Section 1, observational data relevant to galaxy evolution continue to grow rapidly in scale. Consequently, future studies require compact descriptions that make maximal use of the available information.

To utilize the galaxy manifold more effectively and to quantify it, we focus on a class of methods known as dimensionality reduction. Specifically, we apply manifold learning in order to elucidate the dependence of the galaxy manifold on the (presumably small number of) physical quantities that govern the physics of galaxy evolution. Using this approach, we attempt a new quantification of galaxy evolution that is fundamentally different from classical first-principle theoretical constructions in astronomy. Another major advantage of a quantitative representation of the galaxy manifold is that it enables the direct estimation of observational quantities, such as missing luminosities for observed objects, and physical quantities such as the SFR and stellar mass $M_{*}$ based on positions on the manifold. This becomes possible by mapping the galaxy manifold back into the luminosity space. For this inverse mapping, it is convenient to describe distances on the manifold using the metric of the original multi-wavelength luminosity space [19]. Astronomical observations often involve challenging data analyses at the detection limit, and a quantitative representation of the galaxy manifold that can be used for prediction, estimation, and interpolation of observables will become a powerful tool for future astronomical research.

### 3.2. Manifold Learning

In manifold learning, the data are regarded as a finite set of points $\left\{y_{i}\right\}\;\left(i=1,\ldots ,N\right)$ randomly sampled from a smooth *d*-dimensional manifold M endowed with a metric defined by the geodesic rather than in the relativistic sense. Here, the notion of a geodesic is used in the sense of Riemannian geometry *in data space*. That is, a geodesic denotes a curve that locally minimizes distance with respect to the induced metric on the data manifold. This is conceptually different from the geodesics in general relativity, which describe time-like or null worldlines extremizing proper time in a pseudo-Riemannian spacetime. In the present context, geodesics are purely mathematical objects defined within an inferred Riemannian structure distance $d^{M}$. These data points are embedded, via a smooth mapping ψ, into a feature (or input) space $X=R^{n}\;\left(d\ll n\right)$ equipped with the Euclidean metric ${∥\cdot ∥}_{X}$. Denoting the embedded data points in the feature space by $\left\{x_{i}\right\}\;\left(i=1,\ldots ,N\right)$, the embedding map is given by ψ:M⟶X, and a point $y_{i}\in M$ on the manifold can be written as

(6) $$\begin{array}{c} y_{i}=\psi^{-1}\left(x_{i}\right),\;x_{i}\in X. \end{array}$$

The objective of manifold learning is, given the set of (input) data points $\{x_{i}\}\in X$, to infer the explicit forms of the manifold M and the mapping ψ and to reconstruct the original data points $\{y_{i}\}\in M$. When a manifold learning algorithm is applied to the input data points, the dataset in $R^{n}$ is mapped into a low-dimensional space $R^{d}\;\left(d\ll n\right)$ while preserving relationships among neighboring points. That is,

(7) $$\begin{array}{c} x_{i}\mapsto {\hat{y}}_{i}={\left(\psi_{1}^{-1}\left(x_{i}\right),\ldots ,\psi_{d}^{-1}\left(x_{i}\right)\right)}^{⊤}\in R^{\hat{d}} \end{array}$$

yields an estimate $\left\{{\hat{y}}_{i}\right\}\subset R^{\hat{d}}$ of the original points $\left\{y_{i}\right\}\subset R^{d}$. Here, *⊤* denotes the transpose of a vector. This approach reduces the dimensionality of the data based on the so-called *manifold hypothesis*, which assumes that data in a high-dimensional feature space are distributed on a low-dimensional submanifold (e.g., [20]) and is therefore classified as a form of nonlinear dimensionality reduction. Ideally, the intrinsic dimension *d* of the manifold could also be estimated from the data via $\hat{d}$. In practice, however, we fix $\hat{d}$ and select the optimal $\hat{h}$ based on several criteria (see Section 4.1).

Early studies related to manifold learning date back to sporadic work in the 1990s, but the field gained significant momentum following the publication of two seminal papers [21,22]. Manifold learning algorithms are capable of “unfolding” manifolds with complex geometries in feature space and providing local coordinate systems on them [21,22]. A key requirement is that the connectivity between data points after dimensionality reduction faithfully reflects the connectivity of the original data points in the high-dimensional space; achieving this requires the algorithm to learn the shape of the data. This is the origin of the term “manifold learning.”

While linear methods such as classical PCA are effective at capturing the global structure of data, nonlinear methods are particularly powerful in representing local structures. On the other hand, because many nonlinear techniques focus on preserving local neighborhood relationships, they may fail to retain global structure. Therefore, when applying manifold learning, it is essential to choose an appropriate algorithm depending on the specific objective. It should also be noted that the coordinate systems provided by manifold learning are not guaranteed to possess intuitive or physical meaning (e.g., [23]).This issue is discussed further in Section 4.

### 3.3. *Isomap* and *UMAP* Algorithms

In this study, we adopt Isomap (isometric feature mapping: [22]) and UMAP (uniform manifold approximation and projection [24,25]) as manifold learning algorithms. Our goal is to quantify the dependence of galaxy evolution on physical quantities, and it is therefore necessary that structures that are connected in the original high-dimensional feature space are mapped to connected structures on the manifold. Both algorithms have the property of preserving the connectivity of the original data-point distribution and are thus ideal choices for our purpose. These are summarized as follows:

Isomap: Metric-preserving and density-preserving;
UMAP: Topology-preserving and noise-robust.

For these computations, we used the Python package scikit-learn (v1.X) [26].

#### 3.3.1. Isomap

The Isomap algorithm assumes that a smooth manifold M is a geodesically convex region of $R^{d}\;\left(d\ll n\right)$ and that the embedding map ψ:M⟶X is an isometry. We first define geodesic convexity as follows [22].

**Definition 1** (Geodesically convex)**.** *Let (M,g) be a Riemannian manifold. A subset U⊂M is said to be geodesically convex if, for any two points in U, there exists a unique shortest geodesic contained in U that connects them.*

A geodesically convex Riemannian manifold is also a metric space that is convex with respect to the geodesic distance.

Accordingly, the assumptions of Isomap can be stated as follows:

**Convexity**  M is a geodesically convex subset of $R^{d}$.
**Isometry**    The geodesic distance is preserved under the map ψ. For any two points $y,y^{\prime }\in M$ on the manifold, the geodesic distance between them is equal to the Euclidean distance between the corresponding embedded points x=ψ(y) and $x^{\prime }=\psi \left(y^{\prime }\right)$ in $R^{n}$, i.e.,
  (8) $$\begin{array}{c} d^{M}\left(y,y^{\prime }\right)={∥x-x^{\prime }∥}_{X}\;. \end{array}$$

Isomap is an algorithm that generalizes multidimensional scaling (multidimensional scaling: MDS) by adopting the assumptions that M is a geodesically convex region and that ψ is an isometry. MDS is a method that seeks a lower-dimensional subspace in which the data points are distributed while preserving the Euclidean distances between pairs of data points. MDS is a linear dimensionality reduction method and does not work well on curved manifolds. By extending the spirit of MDS, Isomap approximates the geodesic distances on M between all pairs of data points, thereby preserving the global geometric structure of a nonlinear manifold as much as possible. In this sense, Isomap is local in that it relies on neighborhood relations, while at the same time it is global in that it aims to preserve the overall geometry.

The Isomap algorithm consists of three steps:

1. **Nearest-neighbor search**
  Choose an integer *K* or ϵ>0. Compute the distances between all pairs of data points $x_{i},x_{j}\in X,\;\left(i,j=1,\ldots ,n\right)$ in the feature space X:
  (9) $$\begin{array}{c} d_{ij}^{X}\equiv d^{X}\left(x_{i},x_{j}\right)={∥x-x^{\prime }∥}_{X}\;. \end{array}$$
  As the distance measure, the Euclidean distance is typically used. The neighboring points on M are defined by connecting points up to the *K*-nearest neighbors or all points within a ball of radius ϵ. The performance of Isomap is determined by the choice of *K* or ϵ. For an efficient neighborhood search, Isomap uses sklearn.neighbors.BallTree.
2. **Computation of graph distances**
  For the input data points $\left\{x_{i}\right\}\;\left(i=1,\ldots ,N\right)$, construct a weighted neighborhood graph G=G(V,E). The vertex set V consists of the data points $\left\{x_{1},\ldots ,x_{N}\right\}$, and the edge set E consists of edges $e_{ij}$ that represent neighborhood relations between data points. Each edge $e_{ij}$ is assigned a weight $w_{ij}$ corresponding to the distance $d_{ij}^{X}$ between the two points. If two points $x_{i}$ and $x_{j}$ are not directly connected by an edge, the weight is set to ∞.
  The geodesic distances on M between pairs of points are then estimated by the graph distances $d_{ij}^{G}$ on G. The graph distance $d_{ij}^{G}$ is defined as the length of the shortest path between the two vertices on the graph G. Two points that are not neighbors are connected via the shortest path that links nearest neighbors, and the path length is given by the sum of the corresponding weights. This length provides an approximation to the geodesic distance between the two distant points.
  If the data points are sampled from a probability distribution defined on the manifold M, then, for a flat manifold, the graph distance $d^{G}$ converges to the geodesic distance $d^{M}$ as N⟶∞ [27]. Efficient algorithms for this purpose include the Floyd–Warshall algorithm [28,29] and Dijkstra’s algorithm [30]. The former is known to be effective when the graph is dense, whereas the latter is effective when the graph is sparse (e.g., [15]).
3. **Spectral embedding via MDS**
  Consider the distance matrix $D^{G}\equiv \left(d_{ij}^{G}\right)$, an N×N symmetric matrix. Applying classical MDS to $D^{G}$, we reconstruct a *d*-dimensional space Y such that the geodesic distances between data points on the manifold M are preserved as faithfully as possible. Let $S^{G}\equiv \left({\left(d_{ij}^{G}\right)}^{2}\right)$ be the N×N symmetric matrix for which its entries are the squared graph distances. This matrix is double-centered as
  (10) $$\begin{array}{c} K_{N}^{G}=-\frac{1}{2}HS^{G}H\;, \end{array}$$
  (11) $$\begin{array}{c} H\equiv I_{N}-\frac{1}{N}1_{N}\;. \end{array}$$
  Here, $I_{N}$ denotes the (N×N) identity matrix, and $1_{N}$ denotes the (N×N) symmetric matrix with all entries equal to 1.
4. Choose the embedding vectors $\left\{{\hat{y}}_{i}\right\}$ so as to minimize $∥K_{N}^{G}-K_{N}^{Y}∥$. Here,
  (12) $$\begin{array}{c} K_{N}^{Y}=-\frac{1}{2}HS^{Y}H\;. \end{array}$$
  with $S^{Y}=\left({\left(d_{ij}^{Y}\right)}^{2}\right)$ and $d_{ij}^{Y}=∥y_{i}-y_{j}∥$ being the Euclidean distance between $y_{i}$ and $y_{j}$. If we perform an eigendecomposition of $K_{N}^{G}$ using the eigenvalue matrix $\Lambda =\mathrm{diag}(\lambda_{1},\ldots ,\lambda_{N})$ and the eigenvector matrix $V=(v_{1},\ldots ,v_{N})$, we obtain
  (13) $$\begin{array}{c} K_{N}^{G}=V\Lambda V^{⊤}\;. \end{array}$$
  The optimal solution is given by the eigenvectors $v_{1},\ldots ,v_{d}$ corresponding to the *d* largest eigenvalues $\lambda_{1}\geq \ldots \geq \lambda_{d}$ of $K_{N}^{G}$.
5. The graph G is embedded into the *d*-dimensional subspace Y by the d×N matrix:
  (14) $$\begin{array}{c} Y\equiv \left({\hat{y}}_{1},\ldots ,{\hat{y}}_{N}\right)=\left(\lambda_{1}^{\frac{1}{2}}v_{1},\ldots ,\lambda_{d}^{\frac{1}{2}}v_{d}\right)\;. \end{array}$$

Owing to its construction, Isomap preserves the metric between pairs of points and therefore conserves the “surface density” of data points on the manifold relative to the feature space. That is, regions in which data points are densely populated in the feature space remain dense on the manifold, while sparse regions remain sparse. Because Isomap assumes that the manifold M is a geodesically convex submanifold of Euclidean space and that the mapping ψ is an isometry, it does not perform well in cases where the curvature is too large, the manifold contains holes, or the manifold is non-convex. As a practical issue, when noise is present—i.e., when data points do not lie exactly on the manifold—the performance of Isomap depends on the choice of the neighborhood. If the noise level is not excessively large, Isomap is generally reasonably robust against noise. In this study, we adopt K=5 for the neighborhood size in Isomap.

#### 3.3.2. UMAP

UMAP (uniform manifold approximation and projection) is a relatively recent method proposed in 2018 and is based on differential geometry and algebraic topology. In UMAP, data points that are close in the original feature space are also mapped to nearby points on the manifold. Because of its fast execution time, computational costs are reduced, and dimensionality reduction to manifolds of dimension four or higher is also feasible. UMAP is an algorithm rooted in topological data analysis and Riemannian geometry. It is based on the following three assumptions: (1) The data are uniformly distributed on a Riemannian manifold, (2) the Riemannian metric is locally constant (or can be well approximated as such), and (3) the manifold possesses local connectivity. Under these assumptions, it becomes possible to model manifolds with a fuzzy topological structure. Because the manifold is defined so that data points are distributed as uniformly as possible, the surface density of data points is not preserved in UMAP, in contrast to Isomap. The UMAP algorithm consists of the following three stages:

1. Estimation of the Riemannian manifold;
2. Representation of the distance space using fuzzy topology;
3. Dimensionality reduction.

The core concept of UMAP is its fuzzy topological representation; however, because it is formulated using category theory, a concise description is difficult. Here we therefore restrict ourselves to an outline, and we refer the reader to the relevant literature for details. UMAP is more robust to noise than Isomap owing to its construction. In this study, we adopt K=50 for the neighborhood size in UMAP.

## 4. Results and Discussion

### 4.1. Results: Galaxy Manifolds Derived with Isomap and UMAP

**Figure 2:**
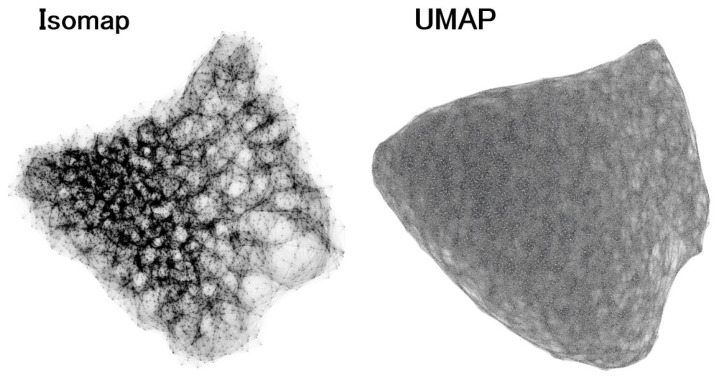
“Unfolded” galaxy manifolds obtained using the manifold learning algorithms Isomap and UMAP. The left and right panels show the manifolds derived from Isomap and UMAP, respectively. The structure of the manifold in this space is much easier to recognize than in Figure 1. Although the overall shapes differ slightly, the characteristic features—such as the distributions of the SFR and stellar mass on the manifold, as shown in the subsequent analysis—are common to both.

The galaxy manifolds obtained with Isomap and UMAP are shown in Figure 2. It is noteworthy that the two different algorithms, Isomap and UMAP, yield qualitatively very similar two-dimensional manifolds (Figure 2). At the same time, the differences between the galaxy manifolds estimated by the two methods are also clearly visible in Figure 2. Because Isomap preserves the density of data points, the resulting manifold exhibits a density structure, i.e., regions of high and low density on the manifold. In contrast, because UMAP constructs the manifold so as to make the density as uniform as possible, the UMAP manifold shows a nearly homogeneous density distribution. In other words, regions of high density in the Isomap manifold appear as such, whereas in the UMAP manifold these regions are expanded in area.

To estimate the dimensionality of the manifolds extracted by Isomap and UMAP, we evaluated them based on reconstruction errors and information criteria. We also attempted an evaluation using the Farahmand–Szepesvári–Audibert (FSA) dimension estimator [31], which is widely used for this purpose. However, upon examination, we found that the estimated dimensionality depends strongly on the choice of neighborhood parameters. For this reason, the FSA results are not used in the discussion of this paper. Further investigation is required to assess the performance of the FSA estimator itself. For both Isomap and UMAP, the reconstruction error does not change beyond numerical accuracy when the dimensionality is increased sequentially from two. Moreover, both the Akaike information criterion [32] and the Bayesian information criterion [33] select a dimensionality of two. Taken together with the results in multi-wavelength luminosity space, we conclude that the dimension of the galaxy manifold derived from this dataset is two. Importantly, the preference for a two-dimensional manifold is supported not only by information criteria but also by the clear physical interpretability of the resulting coordinates in terms of stellar mass and SFR (see Section 4).

**Figure 3:**
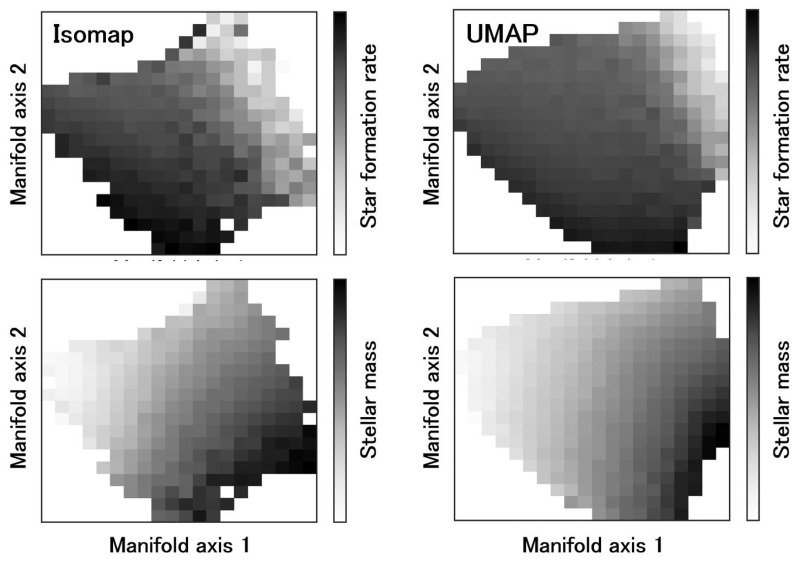
Galaxy manifolds obtained using two different manifold learning algorithms, Isomap and UMAP. The SFR and stellar mass $M_{*}$ are expressed as functions defined on the manifold. The left panel shows the distributions of SFR and stellar mass on the manifold obtained with Isomap. The right panel shows the manifold obtained with UMAP, with the same color coding as in the left panel.

**Figure 4:**
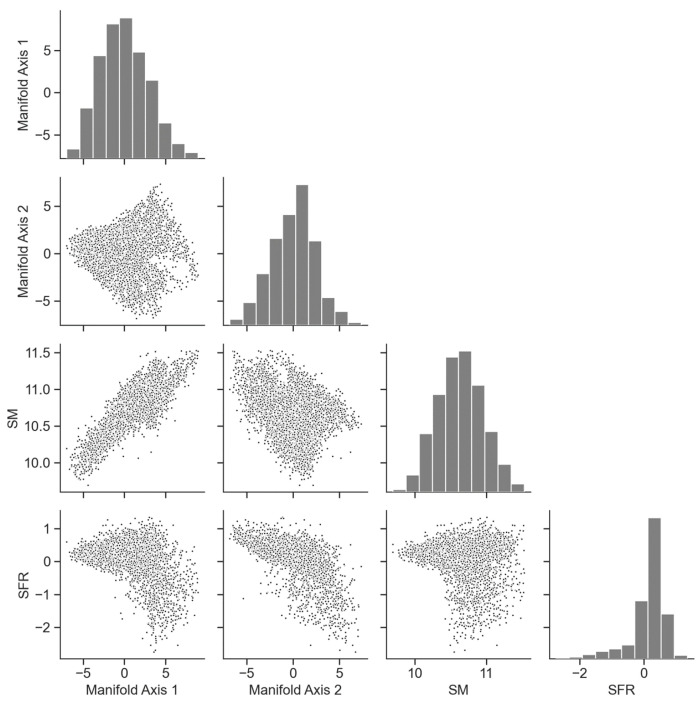
Scatter plots of the coordinates of the two-dimensional galaxy manifold obtained with Isomap and the SFR and stellar mass (SM). Manifold coordinate 1 is strongly correlated with stellar mass, while coordinate 2 is strongly correlated with the SFR.

**Figure 5:**
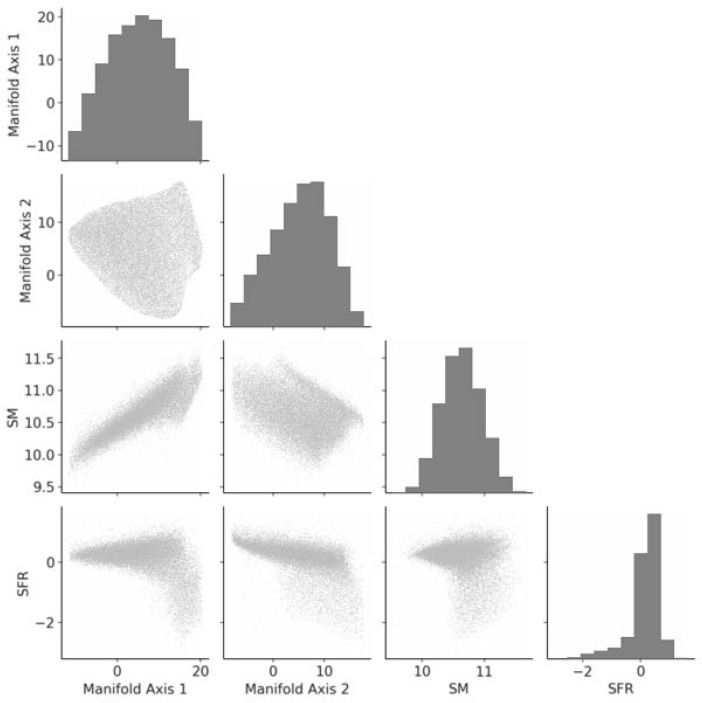
Scatter plots of the coordinates of the two-dimensional galaxy manifold obtained with UMAP and the SFR and stellar mass (SM). As in Figure 4, manifold coordinate 1 is strongly correlated with stellar mass, while coordinate 2 is strongly correlated with the SFR.

To examine how information on galaxy evolution is represented on the galaxy manifold, we compare the SFR and stellar mass as functions defined on the manifold in Figure 3. Figure 4 and Figure 5 show the correlations between the two coordinate axes of the two-dimensional galaxy manifold and the SFR and stellar mass for Isomap and UMAP, respectively. Here, the axes of the Isomap manifold correspond to the eigenvectors associated with the first and second eigenvalues. Although UMAP also yields a two-dimensional manifold, the meanings of its axes are not explicitly defined. The correspondence with the Isomap manifold becomes clear by evaluating the distributions of physical quantities on the manifold. In the following, we therefore discuss the results using axes aligned according to this correspondence.

The behaviors of the SFR and stellar mass are qualitatively very similar in the two figures, suggesting that the estimated manifold structure is robust. This implies that manifold learning has indeed “learned” the essential features of galaxy evolution in multi-wavelength luminosity space. From Figure 4 and Figure 5, we find that manifold coordinate 1 is strongly correlated with stellar mass, while coordinate 2 is strongly correlated with the SFR. We have already seen that the galaxy manifold in optical luminosity space is fundamentally two-dimensional. This means that galaxy evolution at ultraviolet, optical, and near-infrared wavelengths can be fully and sufficiently described by only two physical quantities—the SFR and stellar mass—which represents an important discovery that places strong constraints on theories of galaxy evolution [34].

**Figure 6:**
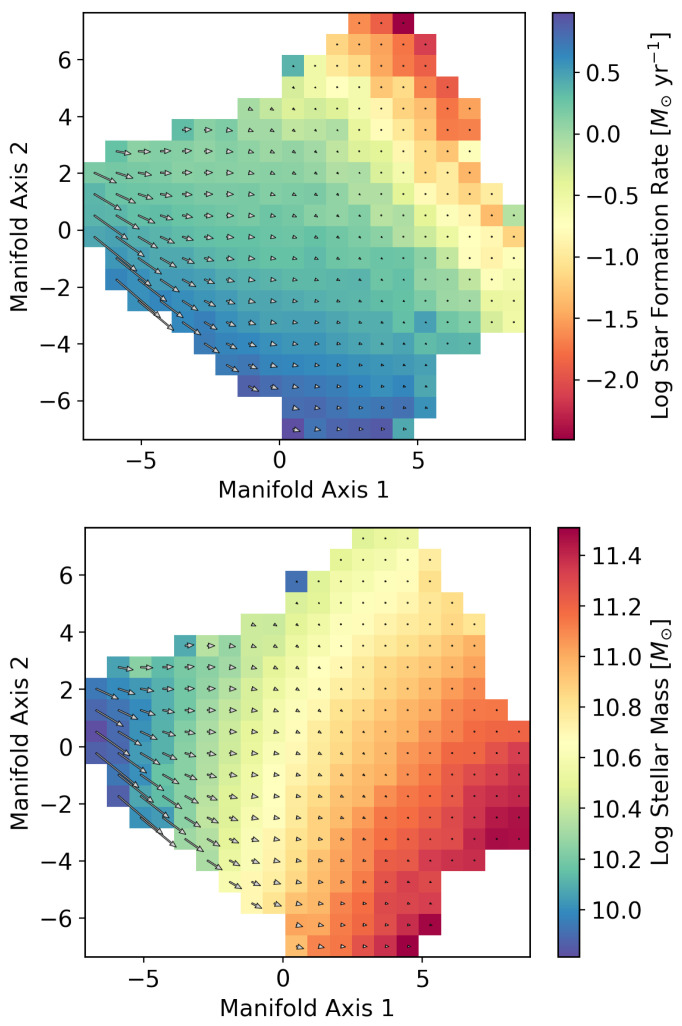
Vector fields of SFR and stellar mass evolution on the Isomap galaxy manifold. The color bar in the upper panel represents the current SFR of galaxies, while that in the lower panel represents the stellar mass.

**Figure 7:**
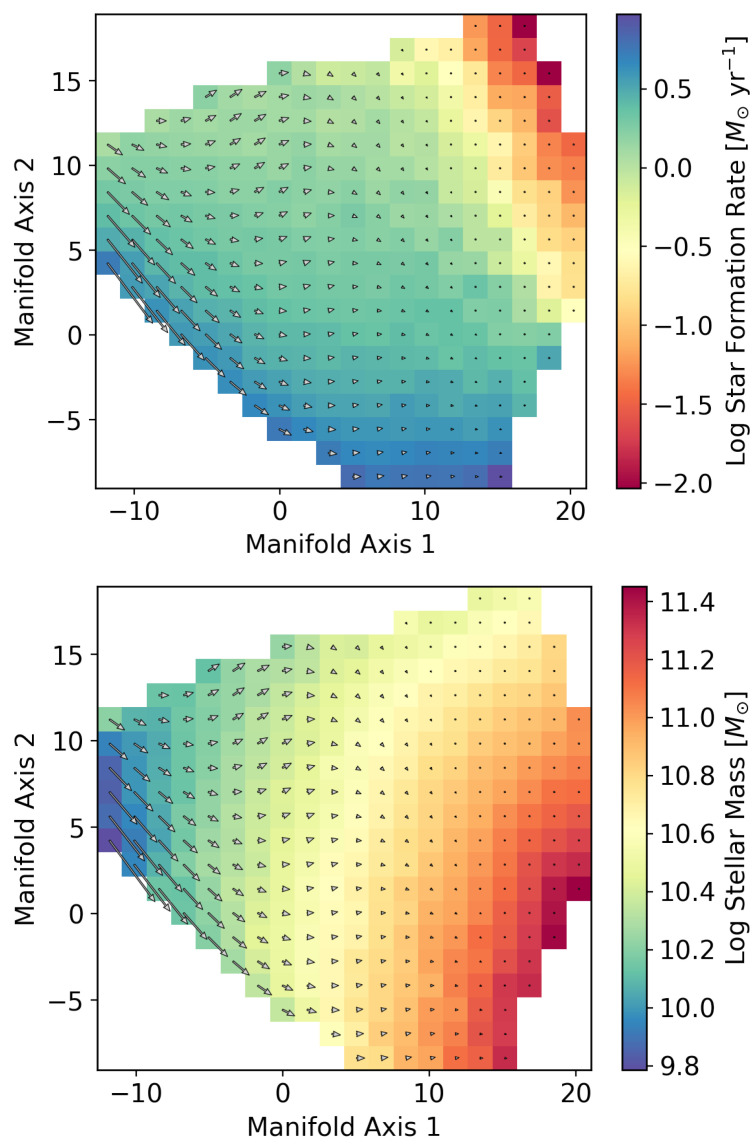
Vector fields of SFR and stellar mass evolution on the UMAP galaxy manifold.

In this way, manifold learning enables us to connect the galaxy manifold with physical quantities such as the SFR and stellar mass. By extending this approach further, it is, in principle, possible to parameterize galaxy evolution directly on the manifold. As stars form, the stellar mass—that is, the accumulated total mass in stars—increases. This is one of the most fundamental aspects of galaxy evolution, and this evolution can be visualized as a vector field on the manifold. The vector fields of star formation are shown in Figure 6 and Figure 7. The “velocity field” of galaxy evolution is clearly visible in these two figures. Low-mass galaxies evolve rapidly, with decreasing star formation rates and increasing stellar masses (from upper left to lower regions in the figures), whereas high-mass galaxies evolve more slowly and remain for a longer time at similar locations on the manifold (upper right).

### 4.2. Galaxy Manifold and Observables

**Figure 8:**
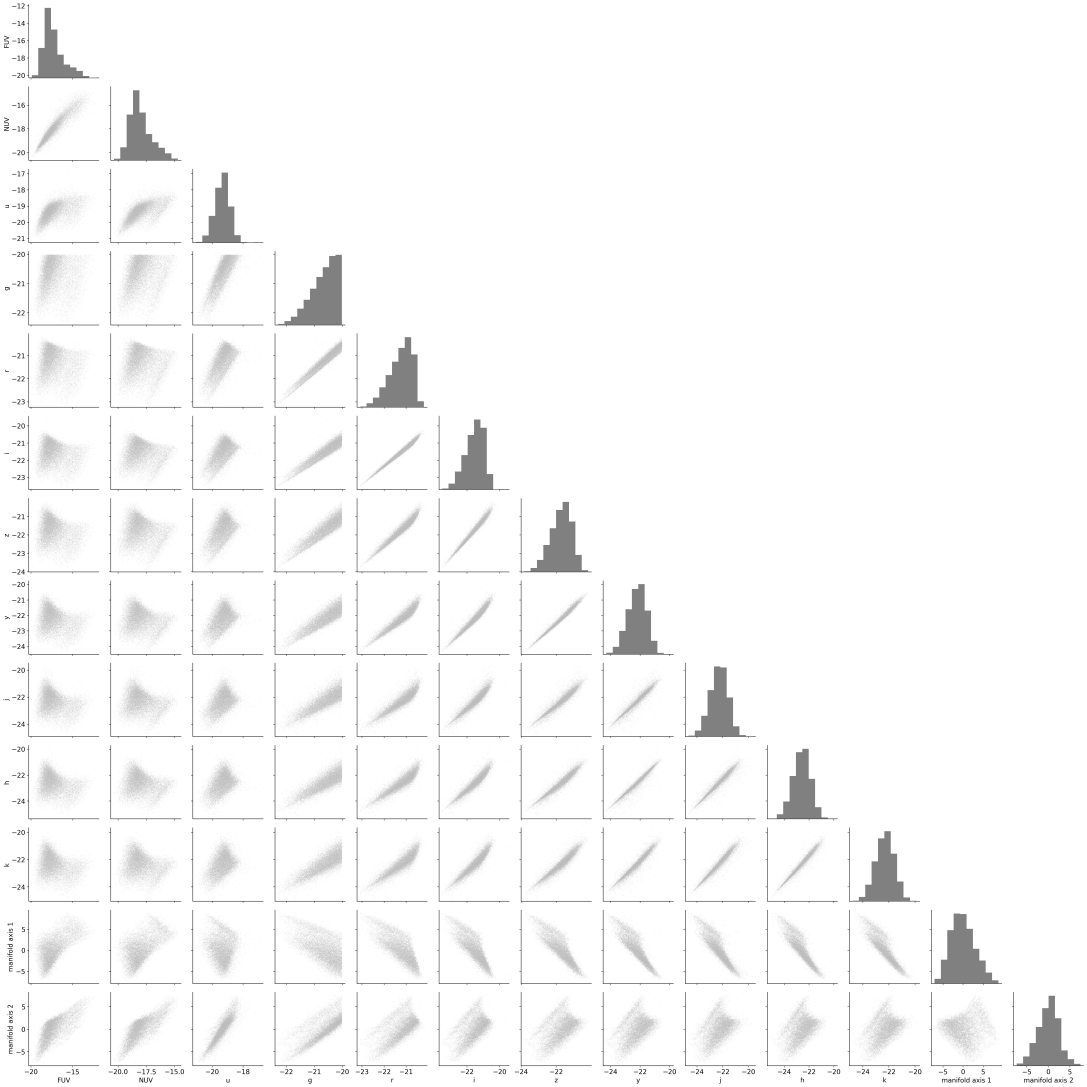
Correlations between galaxy luminosities in each band and galaxy manifold coordinates 1 and 2.

To interpret the galaxy manifold by mapping it back to information in the input space, namely, the multi-wavelength luminosity space, we present pair plots between the manifold coordinates and the observed luminosities in Figure 8. Manifold coordinate 1 is closely correlated with luminosities in the long-wavelength optical to near-infrared regime—specifically the r,i,Y,I,J,H, and *K* bands—which are dominated by contributions from old stellar populations that constitute the backbone of galaxies. In contrast, manifold coordinate 2 is tightly correlated with ultraviolet luminosities (FUV and NUV) and with the short-wavelength optical *u* and *g* bands, and thus, it traces ongoing or very recent star formation activity. Focusing on optical luminosities, we find that they are very tightly correlated with one another. This implies that including multiple luminosities in the optical wavelength range in the analysis does not essentially add independent information about galaxy properties. In contrast, ultraviolet luminosities exhibit nonlinear correlations that are not trivially apparent in scatter plots, indicating that combinations of ultraviolet and optical bands provide fundamental information on the structure of the manifold. Near-infrared band luminosities behave similarly to optical luminosities, but these correlations suggest that additional information on the manifold structure is still present. Therefore, the feature space of galaxies spanned by 11 multi-wavelength luminosities is necessarily represented by a lower-dimensional submanifold. This constitutes the astrophysical basis underlying the discovered two-dimensional galaxy manifold.

### 4.3. From Quantification to Formulation

The remaining challenge is how to describe and interpret the evolutionary trajectories of galaxies on the manifold. This is, of course, not straightforward and requires further investigation. In Cooray et al. [34], we applied a classical theoretical model of galactic chemical evolution. Chemical evolution is a field of galaxy astrophysics that deals with the formation and evolution of elements within galaxies based on stellar evolution theory. The somewhat peculiar term “chemical” evolution originates from the historical use of this theory to analyze the chemical composition of stars and the interstellar medium (ISM) within galaxies. The key physical process is nucleosynthesis produced by nuclear fusion in stellar interiors. We adopt a simple model with mass outflows proposed by Lilly et al. [35]:

(15) $$\begin{array}{cc} M_{*}\left(t_{n+1}\right) & =M_{*}\left(t_{n}\right)+\left(1-r\right)\mathrm{SFR}\left(t_{n}\right)\Delta t\;, \end{array}$$

(16) $$\begin{array}{cc} M_{\mathrm{ISM}}\left(t_{n+1}\right) & =M_{\mathrm{ISM}}\left(t_{n}\right)-\left(1-r+\zeta \right)\mathrm{SFR}\left(t_{n}\right)\Delta t \end{array}$$

**Figure 9:**
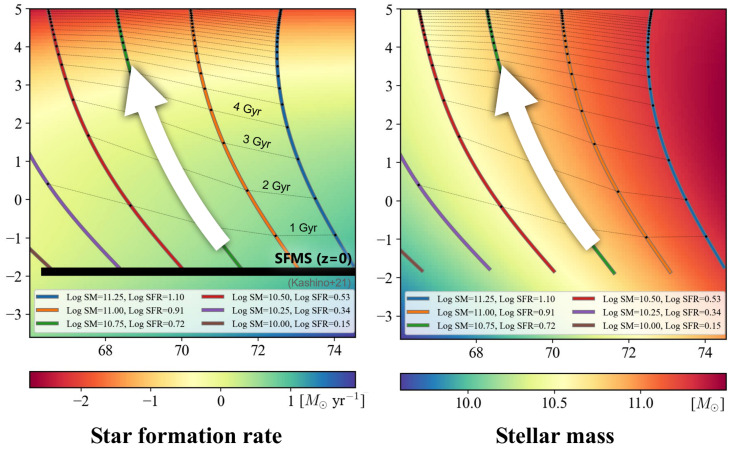
Theoretical evolutionary tracks predicted by a classical chemical evolution model of galaxies (cf. [34]). Thick solid lines show galaxy evolutionary tracks for different initial stellar masses. Thin lines indicate isochrones of galaxy age and are labeled in the left panel (star formation rate space) for clarity; the same isochrones appear in the right panel (stellar mass space) but are unlabeled to avoid overcrowding. SFMS denotes the star-forming galaxy main sequence, an empirical relation for galaxies undergoing continuous star formation.

Here, *r* denotes the returned mass fraction (the fraction of gas returned to the interstellar medium), ζ is the mass-loading factor (the ratio of mass outflow rate to the SFR), and Δt represents the time step [34]. As shown in Figure 9, theoretical evolutionary trajectories of galaxies can be computed from Equations (15) and (16). The interpretation of the vector fields on the galaxy manifold can be obtained by comparing Equations (15) and (16) with Figure 6 or Figure 7. As discussed in Section 4.2 in connection with Figure 8, multi-wavelength luminosities are strongly correlated with one another and can be broadly divided into quantities related to the SFR and the stellar mass $M_{*}$. Nevertheless, as seen in Equation (1), determining the number of independent physical quantities from first principles alone is extremely difficult for complex systems such as those encountered in astrophysics. The range of degrees of freedom anticipated from purely physical considerations is rather broad, and at present, the most effective approach is to constrain this range using dimensional information obtained from data science methods such as manifold learning.

However, the current approach remains unsatisfactory in that the evolutionary equations cannot be uniquely determined directly from the galaxy manifold itself. A more direct interpretation and formulation of the galaxy manifold intrinsically requires more sophisticated methodologies, and we are currently addressing this problem using approaches such as symbolic regression (e.g., [36]).

## 5. Conclusions and Outlook

In this study, we applied manifold learning—an approach that has recently developed within data science—to the distribution of galaxies in a high-dimensional feature space spanned by their multi-wavelength luminosities, and we characterized the evolutionary properties of galaxies. As a result, we discovered a low-dimensional nonlinear structure embedded in the data points within multi-wavelength luminosity space, which we refer to as the *galaxy manifold*. We further found that galaxy evolution in ultraviolet, optical, and near-infrared luminosity space is well described by two parameters on the galaxy manifold: star formation and stellar mass evolution. This result demonstrates the effectiveness of manifold learning for studies of galaxy evolution.

The data used in this study span wavelengths from the ultraviolet to the near-infrared range. Emission in this wavelength range is primarily dominated by stellar radiation, with additional contributions from gas. However, by extending the analysis to wavelengths farther from the optical regime—such as short-wavelength emission including γ-rays and X-rays or mid- and far-infrared wavelengths dominated by dust emission, as well as radio wavelengths that trace atomic and molecular gas emission—additional physical processes can be incorporated. We are currently performing the same analysis in a multi-wavelength luminosity space that includes mid-infrared data, and we have obtained indications that the resulting manifold contains information on dust emission in galaxies. Short-wavelength emission is closely related to high-energy phenomena, and this framework can also be extended to include the evolution of active galactic nuclei, which are powered by accretion onto black holes and produce extremely high-energy radiation.

Although the discussion here has been restricted to multi-wavelength photometric surveys, this methodology can also be applied to spectroscopic survey data. Furthermore, by incorporating not only radiative properties but also the dynamical, structural, and environmental characteristics of galaxies, it becomes possible to address more dynamical physical processes such as galaxy formation, interactions, and mergers, opening a path toward a grand unified theory of galaxy evolution.

In this paper, galaxy evolution has been described as a vector field on the galaxy manifold. If data become available that allow the construction of galaxy manifolds at different cosmic epochs, galaxy evolution could be described not as vectors on a fixed manifold but rather as the evolution of the galaxy manifold itself. With future redshift surveys capable of obtaining sufficiently rich data from the distant Universe, this methodology is expected to become an even more powerful tool.

In this way, manifold learning provides fundamentally new insights into studies of galaxy formation and evolution. Nevertheless, this represents only one of the simplest examples among the many possible applications of manifold learning to physics. Beyond describing physical phenomena, manifold learning has the potential to become a new methodology for the discovery and unification of physical laws more broadly.

## Data Availability

The data used in this study are publicly available from the referenced survey catalogs. No new data were created in this study.

## Appendix A. Basics of Cosmology

### Appendix A.1. The Friedmann–Lemaître–Robertson–Walker Metric and the Scale Factor

General relativity demonstrated that spacetime is characterized by its geometric structure and established differential geometry as the fundamental framework to be employed. In differential geometry, the basic concept used to describe local properties on a manifold is the line element, or metric. The Friedmann–Lemaître–Robertson–Walker (FLRW) metric was proposed to describe the (local) geometric structure of a homogeneous and isotropic spacetime, and it is widely used as a cosmological model. Here, we stress again that the manifold discussed in this work is an emergent structure in data space, defined via similarity relations between galaxies. It is therefore fundamentally distinct from spacetime manifolds in general relativity, which are equipped with Lorentzian metrics. Our construction assumes a Riemannian metric induced from the embedding feature space.

(A1) $$\begin{array}{c} ds^{2}=g_{\mu \nu }dx_{\mu }dx_{\nu }=-c^{2}dt^{2}+a^{2}\left(t\right)\left[\frac{dr^{2}}{1-Kr^{2}}+r^{2}\left(d\theta^{2}+\sin^{2}\theta d\phi^{2}\right)\right] \end{array}$$

Here, $g_{\mu \nu }$ denotes the metric tensor, *t* is cosmic time, and r,θ, and ϕ are the radial and angular coordinates in polar coordinates. The quantity *K* represents the Gaussian curvature, and a(t) is the scale factor. The scale factor describes the change in the spatial scale due to cosmic expansion and is conventionally normalized in cosmology as $a_{0}\equiv a\left(t_{0}\right)=1$, where $t_{0}$ is the present age of the Universe. Here, we adopt a definition in which *K* has dimensions of ${\left(\mathrm{length}\right)}^{-2}$. In this case, *r* has dimensions of length, and a(t) is dimensionless.

By introducing the scale factor, one can separate the net change in distances between objects due to their intrinsic motion from the “trivial” change caused by cosmic expansion. That is, if the position vector representing distance is denoted by $\vec{r}$, it can be written as

(A2) $$\begin{array}{c} \vec{r}=a\left(t\right)\vec{x}\;. \end{array}$$

The vector $\vec{x}$ represents distances with the effect of cosmic expansion removed, and it is referred to as the comoving coordinate.

### Appendix A.2. Cosmological Redshift

Consider a situation in which light emitted at a past time $t=t_{\mathrm{em}}$ from comoving coordinates $\left(r,\phi ,\theta \right)=\left(r_{e},0,0\right)$ reaches an observer located at the origin (r,ϕ,θ)=(0,0,0) at time $t=t_{0}$. In relativity, light propagates along null geodesics. A null geodesic is a path that satisfies $ds^{2}=0$ and may be regarded as the trajectory realized by the principle of least action. Using Equation (A1), distance and time are related by

(A3) $$\begin{array}{c} \frac{cdt}{a(t)}=\frac{dr}{\sqrt{1-Kr^{2}}}\;. \end{array}$$

To discuss the effect of cosmic expansion on light, we transform the variable *r* to the coordinate distance χ via

(A4) $$\begin{array}{c} \chi \equiv \int_{0}^{r_{\mathrm{em}}}\frac{dr}{\sqrt{1-Kr^{2}}}, \end{array}$$

and the time variable *t* to the conformal time η via

(A5) $$\begin{array}{c} \eta \equiv \int^{t}\frac{dt^{\prime }}{a(t^{\prime })}. \end{array}$$

The equation governing the propagation of light then becomes

(A6) $$\begin{array}{c} c(\eta_{\mathrm{obs}}-\eta_{\mathrm{em}})=\chi , \end{array}$$

where *c* is the speed of light, and $\eta_{\mathrm{obs}}$ denotes the time at which light reaches the observer.

Suppose that light emitted at times $\eta =\eta_{0}$ and $\eta =\eta_{0}+\delta \eta_{0}$ reaches the observer at times $\eta_{1}$ and $\eta_{1}+\delta \eta_{1}$, respectively. Because the right-hand side of Equation (A6) is independent of η, we obtain

(A7) $$\begin{array}{c} \delta \eta_{0}=\delta \eta_{1}, \end{array}$$

or equivalently,

(A8) $$\begin{array}{c} \frac{\delta t_{0}}{a(t_{0})}=\frac{\delta t_{1}}{a(t_{1})}. \end{array}$$

Since the phase of light is conserved over the time intervals $\delta t_{0}$ and $\delta t_{1}$, we obtain

(A9) $$\begin{array}{c} a\left(t_{0}\right)\nu_{0}=a\left(t_{1}\right)\nu_{1}⟺\frac{\lambda_{0}}{a(t_{0})}=\frac{\lambda_{1}}{a(t_{1})}, \end{array}$$

where ν denotes the frequency of light, which is related to the wavelength by λν=c.

In an expanding Universe, $a\left(t_{0}\right)>a\left(t_{1}\right)$, so the wavelength observed at the present time is longer than that at emission, i.e., $\lambda_{0}>\lambda_{1}$. This stretching of wavelength due to cosmic expansion is referred to as cosmological redshift. The redshift *z* is defined as

(A10) $$\begin{array}{c} z\equiv \frac{\lambda_{0}-\lambda_{1}}{\lambda_{1}}=\frac{a(t_{0})}{a(t_{1})}-1. \end{array}$$

Here, we normalize the scale factor such that $a(t_{0})=1$. With this normalization, the relation between the scale factor a(t) and the redshift *z* becomes

(A11) $$\begin{array}{c} z=\frac{1}{a(t)}-1\;, \end{array}$$

or equivalently,

(A12) $$\begin{array}{c} a\left(t\right)=\frac{1}{1+z}. \end{array}$$

### Appendix A.3. Friedmann Equations and Cosmological Parameters

With the preparation above and the Einstein equation, one can derive the “equations of motion” that describe an expanding Universe. The Einstein equation is the fundamental equation of general relativity that relates the curvature of spacetime to the energy density, and it is written as

(A13) $$\begin{array}{c} R^{\mu \nu }-\frac{1}{2}Rg^{\mu \nu }+\Lambda g^{\mu \nu }=\frac{8\pi G}{c^{4}}T^{\mu \nu }. \end{array}$$

Here, $R^{\mu \nu }$ is the Ricci tensor, $R\equiv {R^{\alpha }}_{\alpha }$ is its trace (the Ricci scalar), *G* is Newton’s gravitational constant, and Λ is the cosmological constant. Details are deferred to the literature on general relativity, but the Ricci tensor is a second-rank covariant tensor that expresses spacetime curvature as a function of the metric $g_{\mu \nu }$ and its derivatives (e.g., [37]). For the FLRW metric, the equations of motion for spacetime become

(A14) $$\begin{array}{cc} {\left(\frac{\dot{a}}{a}\right)}^{2} & =\frac{8\pi G\rho }{3}-\frac{c^{2}K}{a^{2}}+\frac{c^{2}\Lambda }{3} \end{array}$$

(A15) $$\begin{array}{cc} \frac{\ddot{a}}{a} & =-\frac{4\pi G}{3}\left(\rho +\frac{3p}{c^{2}}\right)+\frac{c^{2}\Lambda }{3}\;. \end{array}$$

These are known as the Friedmann equations. Here, ρ denotes the energy density of the Universe, and *p* denotes the pressure. Equation (A14) corresponds to the 00 component of the Einstein equation (Equation (A13)), while Equation (A15) is obtained from its trace.

It is useful to introduce the following quantities, known as cosmological parameters, which allow a more explicit description of the physical dependence of cosmic expansion:

- Hubble parameter:
  (A16) $$\begin{array}{c} H\left(t\right)\equiv \frac{\dot{a}\left(t\right)}{a(t)}\;. \end{array}$$
  Although this quantity is time dependent, observationally its present value $H_{0}$ is often used. The dimensionless parameter $h=H_{0}/100$ is also frequently employed.
- Density parameter:
  (A17) $$\begin{array}{c} \Omega_{M}\left(t\right)\equiv \frac{\rho (t)}{\rho_{c}\left(t\right)}\equiv \frac{8\pi G\rho (t)}{3H^{2}\left(t\right)}\;, \end{array}$$
  Here, $\rho_{c}$ is the critical density. In the present Universe, it is measured to be $\rho_{c}=1.88\times 10^{-29}h^{2}\;\left[{g\;cm}^{-3}\right]$.
- Dimensionless cosmological-constant parameter:
  (A18) $$\begin{array}{c} \Omega_{\Lambda }\left(t\right)\equiv \frac{c^{2}\Lambda }{3H^{2}\left(t\right)}. \end{array}$$
- Curvature parameter:
  (A19) $$\begin{array}{c} \Omega_{K}\left(t\right)\equiv -\frac{c^{2}K}{a{\left(t\right)}^{2}H^{2}\left(t\right)}. \end{array}$$

Using Equations (A16)–(A19), Equation (A14) can be written as

(A20) $$\begin{array}{c} \Omega_{M}\left(t\right)+\Omega_{\Lambda }\left(t\right)+\Omega_{K}\left(t\right)=1. \end{array}$$

Because this relation holds at any cosmic time *t*, at the present age of the Universe $t_{0}$, it reduces to

(A21) $$\begin{array}{c} \Omega_{M0}+\Omega_{\Lambda 0}+\Omega_{K0}=1. \end{array}$$

As mentioned in Section 1, the cosmological parameters adopted in this paper, $h=H_{0}/100\;\left[\mathrm{km}\;s^{-1}\;{\mathrm{Mpc}}^{-1}\right]=0.7$, $\Omega_{\Lambda 0}=0.7$, $\Omega_{M0}=0.3$, and curvature parameter $\Omega_{K0}=0$, are supported by the latest precision cosmological observations and indicate a spatially flat Universe undergoing accelerated expansion driven by the cosmological constant (or dark energy) (e.g., [38]).

## Appendix B. Magnitude

The energy passing through a unit area of a detector per unit time is called the flux, and the amount per unit frequency (or per unit wavelength) is called the flux density. In astronomy, instead of using the flux or flux density directly, it is customary to use the magnitude, defined as a number proportional to −2.5 times the logarithm of these quantities. This is expressed as

(A22) $$\begin{array}{c} m_{\nu \mathrm{obs}}=-2.5\log_{10}S_{\nu_{\mathrm{obs}}}+\mathrm{constant}\;. \end{array}$$

Historically, the constant appearing in the definition of magnitude was chosen such that Vega (α Lyrae) has magnitude zero. However, this definition has no physical basis and has long been a source of confusion (e.g., [39]).

The AB magnitude system was introduced by Oke and Gunn [40] to provide a systematic and physically motivated conversion from flux density to magnitude. The AB magnitude is defined as

(A23) $$\begin{array}{c} m_{\mathrm{AB},\nu_{\mathrm{obs}}}=-2.5\log_{10}S_{\nu_{\mathrm{obs}}}-48.60\;. \end{array}$$

Here, the unit of $S_{\nu }$ is $\left[\mathrm{erg}\;s^{-1}\;{\mathrm{cm}}^{-2}\;{\mathrm{Hz}}^{-1}\right]$. The subscript “obs” indicates that the frequency is the value observed in the rest frame of the Earth. The subscript “em” denotes the frequency in the rest frame of the galaxy at the time of emission. Thus, using the cosmological redshift *z*, we have $\nu_{\mathrm{em}}=\left(1+z\right)\nu_{\mathrm{obs}}$. Note that the constant −48.60 does not depend on either the observed frequency ν or the wavelength λ. Accordingly, the relation between the absolute magnitude $M_{\mathrm{AB},\nu }$ and the monochromatic luminosity $L_{\nu }$ is given by

(A24) $$\begin{array}{cc} M_{\mathrm{AB},\nu_{\mathrm{em}}} & =m_{\mathrm{AB},\nu_{\mathrm{obs}}}-25-5\log_{10}d_{L}\left(z\right)\\ \; & =-2.5\log_{10}\left[\frac{\left(1+z\right)L_{\nu_{\mathrm{em}}}}{4\pi {d_{L}\left(z\right)}^{2}}\right]-48.60-25-5\log_{10}d_{L}\left(z\right)\\ \; & =-2.5\log_{10}\left[\left(1+z\right)L_{\nu_{\mathrm{em}}}\right]-48.60-25+2.5\log\left(4\pi \right)\;. \end{array}$$

Here,

(A25) $$\begin{array}{c} d_{L}\left(z\right)=\frac{c}{H_{0}}\int_{0}^{z}\frac{dz^{\prime }}{\sqrt{\Omega_{M0}{\left(1+z^{\prime }\right)}^{3}+\Omega_{\Lambda 0}}}\;\left[\mathrm{Mpc}\right] \end{array}$$

is the luminosity distance of the galaxy, and *z* is its redshift. Moreover,

(A26) $$\begin{array}{c} \frac{c}{H_{0}}=3000h^{-1}\;\left[\mathrm{Mpc}\right]\;, \end{array}$$

is the Hubble length, and

(A27) $$\begin{array}{c} 1\;\left[\mathrm{pc}\right]=3.086\times 10^{18}\;\left[\mathrm{cm}\right] \end{array}$$

(e.g., [41]).

However, the effect of redshift is not limited to a simple stretching of the observed wavelength; one must also account for the change in monochromatic luminosity due to the difference between the observed and emitted wavelengths. The flux density per unit frequency can be expressed in terms of the monochromatic luminosity as

(A28) $$\begin{array}{cc} S_{\nu_{\mathrm{obs}}}\;d\nu_{\mathrm{obs}} & =\frac{L_{\nu_{\mathrm{em}}}\;d\nu_{\mathrm{em}}}{4\pi {d_{L}\left(z\right)}^{2}}=\frac{L_{\nu_{\mathrm{obs}}\left(1+z\right)}\;d\left(1+z\right)\nu_{\mathrm{obs}}}{4\pi {d_{L}\left(z\right)}^{2}}=\frac{\left(1+z\right)\;L_{\nu_{\mathrm{obs}}}\;d\nu_{\mathrm{obs}}}{4\pi {d_{L}\left(z\right)}^{2}} \end{array}$$

which yields

(A29) $$\begin{array}{c} S_{\nu_{\mathrm{obs}}}=\frac{\left(1+z\right)\;L_{\nu_{\mathrm{obs}}}}{4\pi {d_{L}\left(z\right)}^{2}} \end{array}$$

Accordingly, the flux density per unit wavelength $S_{\lambda }$ is given by

(A30) $$\begin{array}{c} S_{\lambda_{\mathrm{obs}}}=\frac{L_{\lambda_{\mathrm{obs}}/\left(1+z\right)}}{4\pi {d_{L}\left(z\right)}^{2}\left(1+z\right)} \end{array}$$

Strictly speaking, the observed flux is the amount of radiation transmitted through a filter that passes only a certain wavelength range. We define the observed band flux at the effective wavelength $\lambda_{0}$ as

(A31) $$\begin{array}{c} S^{\left[\lambda_{0}\right]}\equiv \int S_{\lambda }R_{\lambda }^{\left[\lambda_{0}\right]}d\lambda , \end{array}$$

where $R_{\lambda }^{\left[\lambda_{0}\right]}$ is the wavelength response function of the filter. Using Equation (A30), the observed band flux can be written as

(A32) $$\begin{array}{cc} S^{\left[\lambda_{0}\right]} & =\frac{1}{4\pi {d_{L}\left(z\right)}^{2}\left(1+z\right)}\int L_{\frac{\lambda }{\left(1+z\right)}}R_{\lambda }^{\left[\lambda_{0}\right]}\;d\lambda =\frac{\int L_{\lambda }R_{\lambda }^{\left[\lambda_{0}\right]}\;d\lambda }{4\pi {d_{L}\left(z\right)}^{2}\left(1+z\right)}\frac{\int L_{\frac{\lambda }{\left(1+z\right)}}R_{\lambda }^{\left[\lambda_{0}\right]}\;d\lambda }{\int L_{\lambda }R_{\lambda }^{\left[\lambda_{0}\right]}\;d\lambda } \end{array}$$

Here, the term

(A33) $$\begin{array}{c} \frac{\int L_{\lambda }R_{\lambda }^{\left[\lambda_{0}\right]}\;d\lambda }{4\pi {d_{L}\left(z\right)}^{2}\left(1+z\right)}\equiv {\tilde{S}}^{\left[\lambda_{0}\right]} \end{array}$$

represents the hypothetical band flux that would be obtained in the absence of cosmological redshift. The remaining factor in Equation (A32) expresses the change in flux caused by the fact that different wavelength ranges of the object’s spectrum are observed due to redshift. This is the so-called *k*-correction. Expressed in magnitudes, we obtain

(A34) $$\begin{array}{cc} m_{\lambda_{0}}-M_{\lambda_{0}} & =-2.5\log S^{\left[\lambda_{0}\right]}+2.5\log\left[\frac{4\pi {d_{L}\left(z\right)}^{2}\left(1+z\right)}{4\pi {\left(10\;\left[pc\right]\right)}^{2}}{\tilde{S}}^{\left[\lambda_{0}\right]}\right]\\ \; & =2.5\log\left[{d_{L}\left(z\right)}^{2}\left(1+z\right)\right]-2.5\log\left[\frac{\int L_{\frac{\lambda }{\left(1+z\right)}}R_{\lambda }^{\left[\lambda_{0}\right]}\;d\lambda }{\int L_{\lambda }R_{\lambda }^{\left[\lambda_{0}\right]}\;d\lambda }\right]+25\\ \; & =5\log d_{L}\left(z\right)+2.5\log\left(1+z\right)-2.5\log\left[\frac{\int L_{\frac{\lambda }{\left(1+z\right)}}R_{\lambda }^{\left[\lambda_{0}\right]}\;d\lambda }{\int L_{\lambda }R_{\lambda }^{\left[\lambda_{0}\right]}\;d\lambda }\right]+25 \end{array}$$

The second and third terms in Equation (A34) represent the *k*-correction expressed in magnitudes (often written as *K*-correction) [42]. The second term accounts for the effect of bandpass stretching, while the third term represents the change in the band flux itself.

## Appendix C. Galaxy Star Formation Histories and Optical Spectra

Here, we provide a quantitative explanation of the relation between star formation histories and galaxy optical (from ultraviolet to near-infrared) spectra. For simplicity, we assume that the optical spectrum of a galaxy is composed solely of stellar contributions. Then, the galaxy spectrum $L_{\lambda }\left(t\right)$ at time *t* is given by

(A35) $$\begin{array}{cc} L_{\lambda }\left(t\right) & =\int_{0}^{t}\int_{M_{\mathrm{low}}}^{M_{\mathrm{up}}}\mathrm{SFR}\left(t-\tau \right)\;F_{\lambda ,Z_{\left(t-\tau \right)}}\left(m,\tau \right)\Phi \left(m\right)\;dm\;d\tau \;, \end{array}$$

where $M_{\mathrm{up}}$ and $M_{\mathrm{low}}$ are the upper and lower limits of stellar masses that form, Φ(m) is the initial mass function (IMF), and $F_{\lambda ,Z_{\left(t-\tau \right)}}$ denotes the spectrum of a star of mass *m* and metallicity Z(t−τ). The initial mass function is a quantity proportional to the mass distribution function (probability density function) of stars formed in a stellar population. Here, we adopt the normalization

(A36) $$\begin{array}{c} \int_{M_{\mathrm{low}}}^{M_{\mathrm{up}}}m\Phi \left(m\right)\;dm=1\;\left[M_{⊙}\right] \end{array}$$

That is, Φ(m) specifies how many stars of a given mass are formed per unit of total formed stellar mass. Metallicity is the fraction of the total interstellar medium mass that is in heavy elements produced by nuclear fusion in stellar interiors. Because the stars observed in a galaxy at time *t* were formed at earlier times, the spectrum and metallicity are evaluated at t−τ rather than at *t*, and they are integrated over τ.

In more detailed theoretical models, one must additionally take into account emission from gas and attenuation by dust (small solid particles composed of heavy elements such as carbon and silicon) formed from stars and other processes (e.g., [11]). However, Equation (A35) is sufficient for understanding the main results of this study. Note, however, that this formulation is based on classical galaxy evolution theory, i.e., a framework that assumes an isolated galaxy evolving in time. As discussed in Section 1, galaxies evolve not only through internal star formation but also through growth via mergers. Thus, Equation (A35) can describe only the internal evolution of the fragments that eventually merge, and we emphasize that an approach such as that adopted in this study is required in order to describe the statistical evolution of galaxies.

## Appendix D. Multidimensional Scaling

Multidimensional scaling (MDS) is a method for representing (or visualizing) similarities among data points when a notion of distance between data points is defined. Given a set of data points and pairwise distances between them, MDS embeds the data into a Euclidean space so that the pairwise distances are preserved as faithfully as possible.

Suppose that we have *N* data points. Given a distance matrix containing all pairwise distances and a target dimension *d*, MDS places the *N* points in a *d*-dimensional Euclidean space such that the distances between pairs of points are approximately preserved. Since the target dimension *d* is typically chosen to be smaller than the original data dimension, MDS can be regarded as a dimensionality-reduction method.

Several variants of MDS exist; in this paper, we use *classical MDS*. Classical MDS is also known as principal coordinate analysis (PCoA), Torgerson scaling, or Torgerson–Gower scaling.

Let *N* be the number of data points, and let D be the distance matrix for which its (i,j)-component is the distance $d_{ij}$ between the *i*-th and *j*-th data points. By symmetry and non-degeneracy of the distance, D is an (N×N) symmetric matrix with zero diagonal elements. We further define the matrix $S=({\left(d_{ij}\right)}^{2})$, for which its elements are the squared distances.

Classical MDS outputs *d*-dimensional coordinates $\left(u_{1},\ldots ,u_{N}\right)$ with $u_{i}\in R^{d}$ for the data points. These coordinates are represented as a matrix $U\in R^{d\times N}$ for which its *i*-th column is $u_{i}$. The matrix U is obtained as follows.

1. We first introduce the kernel (Gram) matrix K defined by
  (A37) $$\begin{array}{c} K=U^{⊤}U\in R^{N\times N}. \end{array}$$
2. The kernel matrix K is computed from the distance matrix D as
  (A38) $$\begin{array}{c} K=-\frac{1}{2}HSH, \end{array}$$
  where
  (A39) $$\begin{array}{c} H\equiv I-\frac{1}{N}1 \end{array}$$
  is the centering matrix. Here, I is the identity matrix, and 1 is the matrix for which its elements are all unity. This operation is called *double centering*.
3. We perform the eigenvalue decomposition of the kernel matrix:
  (A40) $$\begin{array}{c} K=V\Lambda V^{⊤}, \end{array}$$
  where $V=({\vec{v}}_{1},\ldots ,{\vec{v}}_{N})$ is the matrix of eigenvectors, and $\Lambda =\mathrm{diag}(\lambda_{1},\ldots ,\lambda_{N})$ is the diagonal matrix of eigenvalues.
  Using these, we define the full coordinate matrix
  (A41) $$\begin{array}{c} U_{\mathrm{full}}=\Lambda^{1/2}V^{⊤}\in R^{N\times N}. \end{array}$$
4. The matrix $U_{\mathrm{full}}$ represents an embedding in an *N*-dimensional space. To obtain a *d*-dimensional embedding, we retain the *d* largest eigenvalues $\lambda_{1},\ldots ,\lambda_{d}$ and their corresponding eigenvectors ${\vec{v}}_{1},\ldots ,{\vec{v}}_{d}$, and we define
  (A42) $$\begin{array}{c} U=\Lambda_{d}^{1/2}V_{d}^{⊤}\in R^{d\times N}, \end{array}$$
  where $\Lambda_{d}=\mathrm{diag}\left(\lambda_{1},\ldots ,\lambda_{d}\right)$ and $V_{d}=\left({\vec{v}}_{1},\ldots ,{\vec{v}}_{d}\right)$.

Classical MDS is used as a core component of the Isomap algorithm described in the main text.

## Appendix E. Uniform Manifold Approximation and Projection (UMAP)

In this section, we describe the UMAP algorithm in more detail. As outlined in the main text, the UMAP algorithm consists of the following three stages.

1. **Estimation of the Riemannian manifold**
  UMAP assumes that the data are uniformly distributed on a Riemannian manifold M. The Riemannian manifold (M,g) on which the data lie is estimated using a *K*-nearest-neighbor graph, similarly to Isomap. The data are given as points in $R^{n}$, so M is regarded as embedded in $R^{n}$, but the metric *g* is estimated separately.
  The estimation of the metric is based on the following lemma [24].
  **Lemma A1** 
  ([24])**.** *Let (M,g) be a Riemannian manifold embedded in $R^{n}$, and let p∈M. For a sufficiently small neighborhood U of p, assume that the metric is locally constant and diagonal: $g_{ij}=\mathrm{const}\times \delta_{ij}$. Let B⊂U be an open ball of radius r centered at p in $R^{n}$. Then, the volume of B with respect to the metric g is*
  (A43) $$\begin{array}{c} \mathrm{Vol}\left(B\right)=\int_{B}\sqrt{\det(g)}\;dx_{1}\ldots dx_{n}=\sqrt{\det(g)}\int_{B}dx_{1}\ldots dx_{n}, \end{array}$$
  *where the second equality follows from the constancy of g on U. Since the last integral is the volume of an n-dimensional Euclidean ball of radius r,*
  (A44) $$\begin{array}{c} \mathrm{Vol}\left(B\right)=\sqrt{\det(g)}\frac{\pi^{n/2}r^{n}}{\Gamma \;\left(\frac{n}{2}+1\right)}. \end{array}$$
  *By adjusting r, one may set $\det\left(g\right)=r^{-2n}$, which implies $g_{ij}=r^{-2}\delta_{ij}$. Hence, distances measured with respect to g are $r^{-1}$ times the Euclidean distances, and for p,q∈M,*
  (A45) $$\begin{array}{c} d^{M}\left(p,q\right)=r^{-1}d^{R^{n}}\left(p,q\right). \end{array}$$
  This lemma shows that distances near a point *p* can be computed from Euclidean distances by choosing an appropriate local neighborhood. Given a point set $Y=\left\{y_{1},\ldots ,y_{N}\right\}\subset R^{n}$ sampled uniformly from M, the local metric scale can be estimated by fixing a constant *K* and choosing the Euclidean radius that contains exactly *K* neighbors.
2. **Fuzzy topological representation of distance spaces**
  UMAP represents the data as a *fuzzy topological representation* [43], which is essentially a weighted graph for which its edge weights encode the strength of connectivity. Here, we provide an informal introduction following McInnes et al. [25].
  (a) **Fuzzy sets**
    A fuzzy set generalizes an ordinary set by allowing the membership function
    (A46) $$\begin{array}{c} \mu (x)\in (0,1] \end{array}$$
    to represent the membership strength of *x* in the set. For a threshold *a*, the associated crisp set is
    (A47) $$\begin{array}{c} S(a)=\{x|\mu (x)\geq a\}. \end{array}$$
  (b) **Simplicial sets**
    A simplicial set S assigns, for each *m*, a set $S_{m}$ of simplices of dimension at most *m*. For example, $S_{0}$ is the set of vertices, and $S_{1}$ includes edges.
  (c) **Fuzzy simplicial sets**
    A fuzzy simplicial set replaces each $S_{m}$ by a fuzzy set. For strength *a*, we write $S_{m}\left(a\right)$ for the set of simplices with a membership strength of at least *a*.
  (d) **FinEPMet, FinReal, and FinSing**
    An extended pseudo-metric space (EPMet) (X,d) allows d(x,y)=∞ and d(x,y)=0 for x≠y. Finite EPMets are denoted FinEPMet.
    The functor FinReal maps a fuzzy simplex $\Delta_{m}\left(a\right)$ to an EPMet
    (A48) $$\begin{array}{c} \mathrm{FinReal}\left(\Delta_{m}\left(a\right)\right)=\left(\left\{x_{0},\ldots ,x_{m}\right\},d_{a}\right), \end{array}$$
    with
    (A49) $$\begin{array}{c} d_{a}\left(x_{i},x_{j}\right)=\left\{\begin{array}{cc} -\log a & i\neq j,\\ 0 & i=j. \end{array}\right. \end{array}$$
    Conversely, FinSing maps an EPMet (Y,d) to a fuzzy simplicial set S=FinSing(Y,d), where
    (A50) $$\begin{array}{c} S_{m}\left(a\right)=\left\{\bar{f\left(x_{0}\right)\ldots f\left(x_{m}\right)}|f\in \mathrm{Hom}\left(\mathrm{FinReal}\left(\Delta_{m}\left(a\right)\right),Y\right)\right\}. \end{array}$$
3. **Dimensionality reduction**
  The fuzzy topological representations of the original data *X* and the low-dimensional embedding $Y=\left\{y_{1},\ldots ,y_{N}\right\}\subset R^{d}$ are compared using a cross-entropy objective. The embedding *Y* is obtained by minimizing this objective, typically using stochastic gradient descent.
